# Predicting hormone receptor status in invasive breast cancer through radiomics analysis of long-axis and short-axis ultrasound planes

**DOI:** 10.1038/s41598-024-67145-z

**Published:** 2024-07-30

**Authors:** Jiangfeng Wu, Lifang Ge, Yinghong Guo, Anli Zhao, Jincao Yao, Zhengping Wang, Dong Xu

**Affiliations:** 1 Department of Ultrasonography, Dongyang People’s Hospital, No. 60 Wuning West Road, Dongyang, Zhejiang China; 2 Department of Ultrasonography, Institute of Basic Medicine and Cancer, Chinese Academy of Sciences, The Cancer Hospital of the University of Chinese Academy of Sciences (Zhejiang Cancer Hospital), Hangzhou, China

**Keywords:** Radiomics, Ultrasound, Hormone receptor, Breast cancer, Cancer, Molecular biology, Endocrinology, Oncology

## Abstract

The hormone receptor (HR) status plays a significant role in breast cancer, serving as the primary guide for treatment decisions and closely correlating with prognosis. This study aims to investigate the predictive value of radiomics analysis in long-axis and short-axis ultrasound planes for distinguishing between HR-positive and HR-negative breast cancers. A cohort of 505 patients from two hospitals was stratified into discovery (Institute 1, 416 patients) and validation (Institute 2, 89 patients) cohorts. A comprehensive set of 788 ultrasound radiomics features was extracted from both long-axis and short-axis ultrasound planes, respectively. Utilizing least absolute shrinkage and selection operator (LASSO) regression analysis, distinct models were constructed for the long-axis and short-axis data. Subsequently, radiomics scores (Rad-scores) were computed for each patient. Additionally, a combined model was formulated by integrating data from long-axis and short-axis Rad-scores along with clinical factors. The diagnostic efficacy of all models was evaluated by the area under the receiver operating characteristic (ROC) curve (AUC). The long-axis and short-axis models, consisting of 11 features and 15 features, respectively, were established, yielding AUCs of 0.743 and 0.751 in the discovery cohort, and 0.795 and 0.744 in the validation cohort. The calculated long-axis and short-axis Rad-scores exhibited significant differences between HR-positive and HR-negative groups across all cohorts (all p < 0.001). Univariate analysis identified ultrasound-reported tumor size as an independent predictor. The combined model, incorporating long-axis and short-axis Rad-scores along with tumor size, achieved superior AUCs of 0.788 and 0.822 in the discovery and validation cohorts, respectively. The combined model effectively distinguishes between HR-positive and HR-negative breast cancers based on ultrasound radiomics features and tumor size, which may offer a valuable tool to facilitate treatment decision making and prognostic assessment.

## Introduction

Breast cancer (BC) remains a significant global health challenge, affecting millions of women and accounting for a substantial number of cancer-related deaths^1^. Among its molecular sub-types, hormone receptor (HR) positive breast cancer represents a predominant form, characterized by the expression of estrogen receptor (ER) and/or progesterone receptor (PR)^2^. These receptors play a critical role in the tumor's pathogenesis and serve as important therapeutic targets for endocrine therapy^3,4^. Early and accurate prediction of HR positive breast cancer is vital for timely diagnosis, appropriate treatment selection, and improved patient outcomes.

The prevailing standard for diagnosing HR positive breast cancer involves histological examination and immunohistochemical analysis^5^. Nevertheless, this procedure involves tissue biopsy, which may elicit discomfort and pose potential risks, rendering it unsuitable for certain patients, particularly those unable to undergo tissue sampling in specific situations. Furthermore, breast cancer is characterized by its intricacy, with tumors demonstrating heterogeneity^6^. Then, relying solely on a single biopsy sample may not adequately represent the entirety of the tumor, thus increasing the risk of misjudging the HR status of the tumor^7,8^. Hence, there is an urgent need for a non-invasive and effective method to accurately identify the HR status of the tumor.

Medical imaging has emerged as a crucial tool in the diagnosis and management of breast cancer, enabling non-invasive evaluation of breast lesions. Among the imaging modalities, Ultrasound (US) has gained popularity due to its safety, affordability, and real-time imaging capabilities. Conventional B-mode US and Color Doppler US provide valuable insights into the characteristics of breast lesions, such as size, shape, margins, as well as blood flow velocity and direction. However, their diagnostic accuracy in distinguishing between HR positive and HR negative breast cancers is limited^9–11^, prompting the need to explore advanced imaging techniques that can improve prediction accuracy.

In recent years, US radiomics, an emerging field in medical imaging, has garnered significant attention for its potential in unlocking hidden information within medical images. US Radiomics involves the high-throughput extraction and analysis of quantitative imaging features^12,13^ representing the shape, intensity, and texture of tumors, which capture subtle patterns and heterogeneity within the tumor microenvironment^14^. These features provide valuable insights into tumor biology, treatment response, and patient prognosis, which can be analyzed by different machine learning methods. By leveraging US radiomics analysis, researchers have demonstrated promising results in various cancer types, including breast cancer, in predicting disease subtypes and treatment responses^15–18^.

Several studies have indicated that multiplane ultrasound imaging holds significant predictive value in various aspects of breast cancer. Specifically, it has demonstrated efficacy in the preoperative prediction of metastatic lymph node burden^19^, contributed to the reduction of unnecessary biopsies for breast lesions^20^, and exhibited favorable predictive value for the sonographic phenotypes associated with molecular subtypes of invasive ductal cancer^21^. Long-axis US plane allows for a comprehensive visualization along the longest axis of the lesion, while short-axis plane captures cross-sectional views, facilitating the assessment of internal tissue characteristics. Hence, long-axis and short-axis US planes may offer comprehensive views of breast lesions, each providing unique information about their spatial distribution and internal architecture. The integration of radiomics analysis with long-axis and short-axis US planes may potentially enhance the diagnostic accuracy and predictive value for HR positive breast cancer.

Therefore, this study aims to investigate the predictive value of radiomics analysis in long-axis and short-axis US planes for HR positive breast cancer. We hypothesize that the integration of radiomics features from both planes will improve the discrimination between HR positive and HR negative breast cancers, providing clinicians with a valuable non-invasive tool for early detection and personalized treatment planning.

## Methods

### Patients

The multicenter study was carried out at two hospitals. The study received approval from the Institutional Review Board of Zhejiang Cancer Hospital (Institute 1) and the Institutional Review Board of Dongyang People's Hospital (Institute 2), which exempted the requirement for written informed consent due to the retrospective nature of the study. Patients who met the inclusion criteria and were admitted between June 2019 and January 2022 at Zhejiang Cancer Hospital were designated as the discovery cohort, while those admitted between August 2021 and May 2023 at Dongyang People's Hospital constituted the validation cohort. Our study was conducted in accordance with the Declaration of Helsinki.

**Figure 1:**
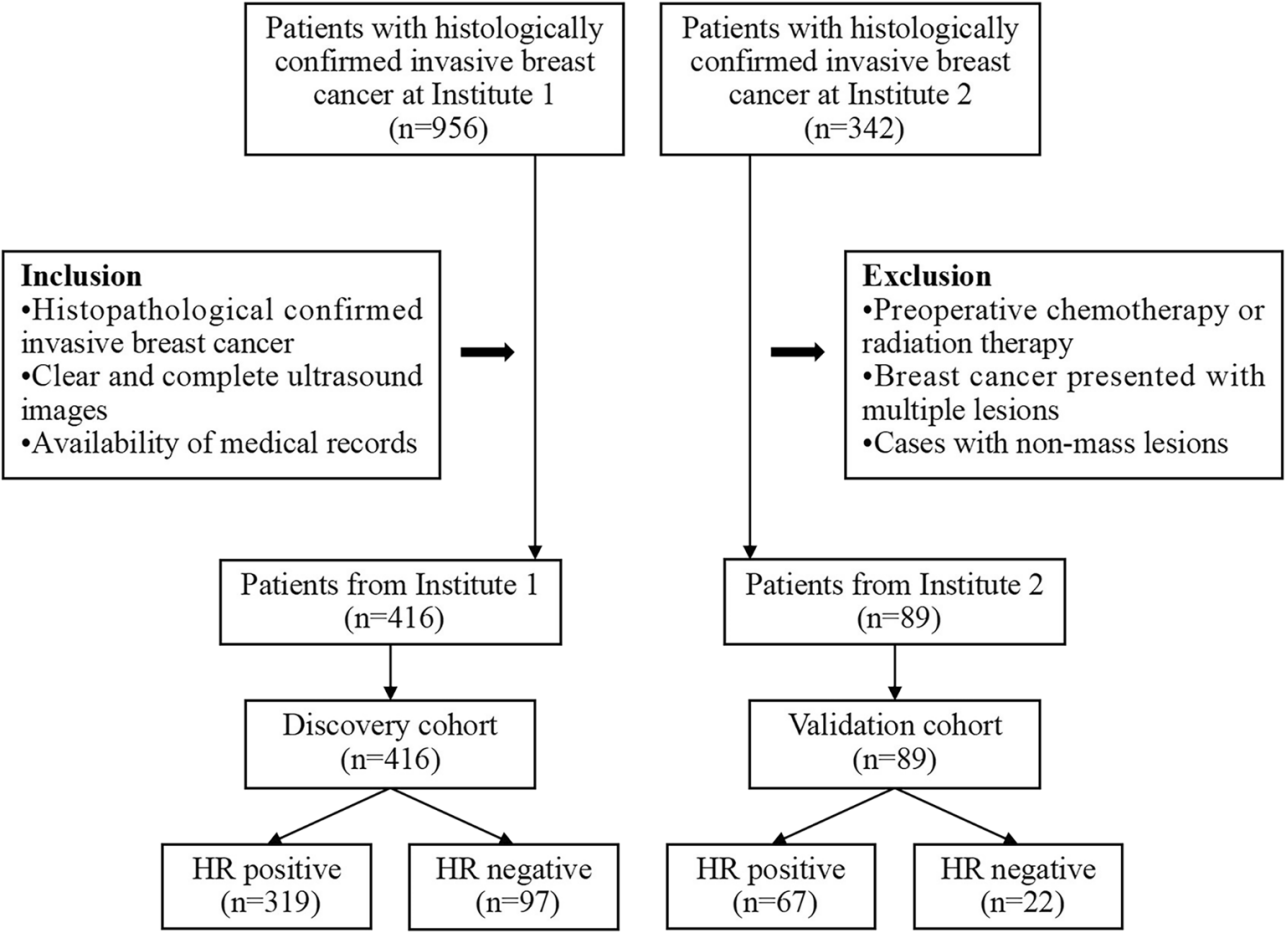
Flowchart illustrating the inclusion and exclusion criteria for the study population. *HR* hormone receptor.

The study's inclusion criteria comprised the following: (1) the postoperative histopathological examination confirmed the presence of invasive breast cancer; (2) clear and complete ultrasound images of the patients' breast tumors; and (3) availability of comprehensive medical records. Conversely, the exclusion criteria included: (1) patients who underwent preoperative chemotherapy or radiation therapy; (2) patients with breast cancer presented with multiple lesions; and (3) cases with non-mass lesions that were difficult to delineate. The flowchart illustrating patient selection and dataset construction is presented in Fig. 1.

Baseline information was systematically collected for each patient, comprising key details such as age, tumor location, US-reported tumor size, Breast Imaging Reporting and Data System (BIRADS) category, US-reported lymph node metastasis, and histopathological features [ER, PR, pathological type, and human epidermal growth factor receptor 2 (HER-2)]. These data were retrieved from clinical records, postoperative pathology reports, and immunohistochemistry analyses, ensuring a comprehensive evaluation and analysis of the patients included in this study.

### Ultrasound imaging acquisition

Ultrasound scans were conducted using four different ultrasound instruments: the LOGIQ E9 system by GE Healthcare, headquartered in Chicago, Illinois, USA; the Siemens Acuson S2000 system by Siemens Healthineers, located in Erlangen, Germany; the Toshiba Aplio 500 system manufactured by Canon Medical Systems in Otawara, Japan; and the Philips EPIQ 5 system by Philips Healthcare, headquartered in Amsterdam, Netherlands. These instruments were equipped with probes of various frequencies, ranging from 5 to 18 MHz, to acquire the largest long-axis and short-axis ultrasound images of the breast lesions. Tumor size was determined as the maximum diameter measured in the long axis of the lesion.

During the scanning process, patients were positioned in a supine or lateral decubitus position, and a water-based gel was applied to the skin surface to improve acoustic coupling. The transducer was placed on the breast region of interest, and real-time B-mode imaging was utilized to visualize the lesion. For each patient, both the long-axis and short-axis planes of the lesion were scanned and captured to ensure comprehensive coverage of the tumor region. The obtained ultrasound images were stored in Digital Imaging and Communications in Medicine (DICOM) format to maintain data integrity and facilitate standardized image analysis across multiple centers. A centralized picture archiving and communication system was utilized to store and manage the DICOM images securely.

### Region of interest identification and segmentation

Following ultrasound imaging acquisition, two experienced Sonographers reviewed the stored DICOM images to identify the regions of interest (ROIs) corresponding to the breast lesions. The long-axis and short-axis ROIs were manually delineated using ITK-SNAP software (Version 3.4.0), respectively, which allowed for precise and consistent segmentation of the tumor area. To ensure accuracy and reliability, each ROI segmentation was carefully reviewed and validated by a senior Sonographer to minimize inter-observer variability.

### Radiomics feature extraction and selection

The original ultrasound images were processed in Python (Version 3.7) using the PyRadiomics package. Isotropic pixel points were ensured through image preprocessing. Image normalization (normalize Scale = 25) and resampling (Resample Pixel Spacing = [1, 1, 1]) were applied during this process. Then radiomics features were extracted from the segmented ROIs by using pyradiomics package, which provided a comprehensive suite of algorithms to quantify various image-based features. These features included first-order statistics, texture features, shape-based characteristics, and wavelet features, all of which contributed to the characterization of the tumor's spatial heterogeneity and texture patterns. To ensure the robustness of the selected features, the intra- and inter-observer agreement in ROI segmentation was assessed using the intraclass correlation coefficient (ICC). A high ICC value > 0.75 indicated strong agreement, enhancing the reliability and reproducibility of the subsequent radiomics analysis process.

Z-score normalization was carried out independently in the discovery and validation cohorts to mitigate the influence of outliers and facilitate the comparison of radiomics features with different magnitudes. To identify the most discriminative features for predicting HR status in the discovery cohort, a rigorous selection process was employed. Firstly, the intra- and inter-observer agreement among Sonographers in ROI segmentation was assessed using the ICC. Subsequently, the Mann–Whitney U test was applied to compare the radiomics features between HR positive and HR negative subgroups, aiming to identify features with significant differences. Lastly, the least absolute shrinkage and selection operator (LASSO) regression was utilized to further select the features that contributed significantly to the predictive model. LASSO regression is a powerful technique to penalize less informative features, effectively promoting the selection of the most discriminative features for building predictive models^22^. By employing LASSO regression, the final set of radiomics features was identified, which played a crucial role in the subsequent model construction for HR status prediction.

### Model construction and validation

In order to identify risk factors associated with HR positive breast cancer, both univariate and multivariate regression analysis were used. In the univariate regression analysis, potential risk factors, including age, tumor location, US-reported tumor size, BIRADS category, and US-reported lymph node metastasis were examined independently. Variables that showed a significant difference (p < 0.05) with HR positive breast cancer in the univariate analysis were then included in the subsequent model construction.

Utilizing the selected radiomics features, logistic regression models were constructed for both the long-axis and short-axis ultrasound planes, and radiomics score (Rad-score) was calculated. Furthermore, a combined model using long-axis and short-axis Rad-scores from both planes and clinical risk factor was established. The logistic regression models were trained using the discovery cohort and validated in the validation cohort to assess their generalization performance. Model performance was evaluated based on various performance metrics, including sensitivity, specificity, accuracy, and the area under the receiver operating characteristic (ROC) curve (AUC).

### Clinical application

**Figure 2:**
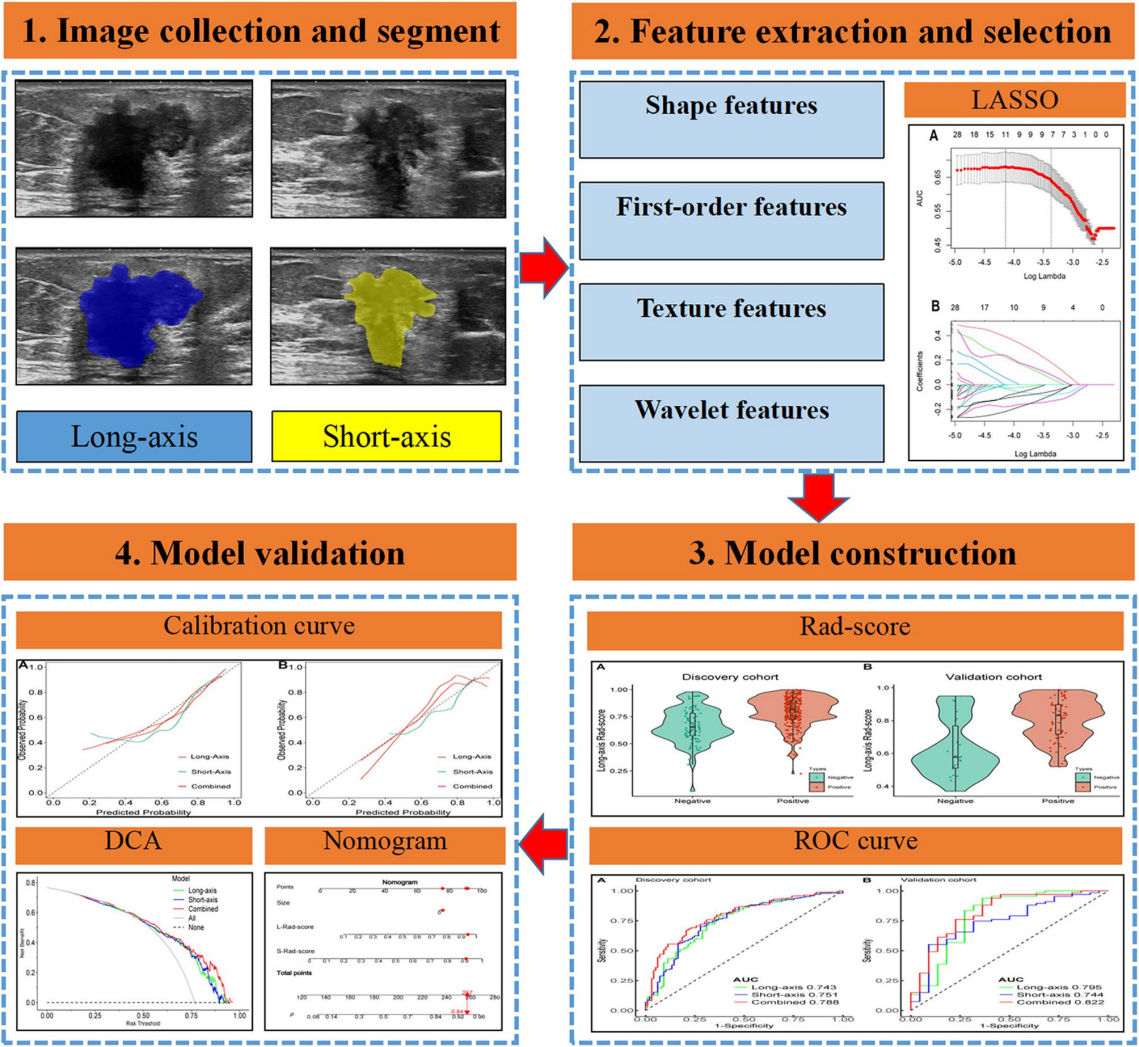
The flowchart depicting the radiomics analysis conducted in this study. *LASSO* least absolute shrinkage and selection operator, *Rad-score* radiomics score, *DCA* decision curve analysis, *ROC* receiver operating characteristic.

To assess its performance and reliability, we first evaluated the model's calibration using a calibration curve. This curve visually compares the predicted probabilities from the model with the actual observed outcomes. A well-calibrated model would show the points on the calibration curve close to the 45-degree reference line, indicating a good agreement between predicted and observed outcomes. Furthermore, we examined the model's discriminative ability through a decision curve analysis (DCA). The DCA allows us to assess the clinical utility of the model by plotting the net benefit against different threshold probabilities. The decision curve provides insights into the model's value in clinical decision-making compared to a "treat-all" or "treat-none" strategy. The complete model architecture is depicted in Fig. 2.

### Statistical analysis

Statistical analyses were conducted using R software (Version 4.1.2). For comparing continuous variables between the HR-positive and HR-negative subgroups, the Student’s t-test was employed for variables with a normal distribution, whereas the Mann–Whitney U test was applied for variables with an abnormal or unknown distribution. For categorical variables, including tumor pathological type, tumor location, and lymph node metastasis, association analyses were performed using either the Chi-square test or Fisher's exact test, depending on sample size and data distribution. A significance level of two-tailed p < 0.05 was deemed as statistically significant.

### Ethics statement

The research involving human participants underwent thorough scrutiny and received official approval from the Institutional Review Board of Dongyang People's Hospital (Approval No. 2024-YX-154) and the Institutional Review Board of Zhejiang Cancer Hospital (Approval No. IRB-2022-548).

## Results

### Patient characteristics

Table 1 provides a comprehensive summary of the patient and tumor characteristics in both the discovery and validation cohorts. It is important to note that no statistically significant differences were observed between these two cohorts, with all p-values > 0.05. This uniformity between the discovery and validation cohorts ensures the reliability of our findings and their potential applicability to a broader patient population.

**Table 1:** Characteristics of patients in the discovery cohort and validation cohort.

Characteristic	Discovery cohort (N = 416)	Validation cohort (N = 89)	P
Age (median)	51.5	54.0	0.233
Right breast tumor (%)	218 (52.4)	49 (55.1)	0.726
Lesion size (median, mm)	20.0	22.0	0.466
ER positive (%)	315 (75.7)	67 (75.3)	1.000
PR positive (%)	262 (63.0)	59 (66.3)	0.628
HER2 positive (%)	95 (22.8)	23 (25.8)	0.581
Pathological type (%)			0.079
IDC	351 (84.4)	75 (84.3)	
ILC	10 (2.4)	6 (6.7)	
Other	55 (13.2)	8 (9.0)	
BIRADS category (%)			0.747
4A	58 (13.9)	12 (13.5)	
4B	100 (24.0)	23 (25.8)	
4C	98 (23.6)	23 (25.8)	
5	160 (38.5)	31 (34.9)	
US-reported LN metastasis (%)	171 (41.1)	29 (32.6)	0.152

*ER* estrogen receptor, *PR* progesterone receptor, *HER-2* human epidermal growth factor receptor 2, *LN* lymph node, *US* ultrasound, *BI-RADS* Breast Imaging Reporting and Data System, *IDC* invasive ductal carcinoma, *ILC* invasive lobular carcinoma.

Table 2 offers a detailed view of the clinicopathological characteristics of patients, categorized into HR positive and HR negative groups. In the discovery cohort, several parameters exhibited notable differences between these groups. Specifically, ER PR, HER-2, and tumor size displayed significant variations. Similar trends were observed in the validation cohort, where ER, PR, pathological type, and tumor size remained statistically significant variations distinguishing between HR positive and HR negative patients.

**Table 2:** Association of patient characteristics with hormone receptor status in the discovery and validation cohorts.

Characteristic	Discovery cohort		p	Validation cohort		p
	HR negative (N = 97)	HR positive (N = 319)		HR negative (N = 22)	HR positive (N = 67)	
Age (median)	53.0	51.0	0.986	54.0	54.0	0.823
Right breast tumor (%)	51 (52.6)	167 (52.4)	1.000	12 (54.5)	37 (55.2)	1.000
Lesion size (median, mm)	25.0	20.0	0.006	28.0	20.0	0.028
ER positive (%)	0 (0.0)	315 (98.7)	< 0.001	0 (0.0)	67 (100.0)	< 0.001
PR positive (%)	0 (0.0)	262 (82.1)	< 0.001	0 (0.0)	59 (88.1)	< 0.001
HER2 positive (%)	51 (52.6)	44 (13.8)	< 0.001	8 (36.4)	15 (22.4)	0.261
Pathological type (%)			0.100			0.017
IDC	76 (78.4)	275 (86.2)		17 (77.3)	58 (86.6)	
ILC	2 (2.1)	8 (2.5)		0 (0.0)	6 (9.0)	
Other	19 (19.6)	36 (11.3)		5 (22.7)	3 (4.5)	
BIRADS category (%)			0.054			0.810
4A	11 (11.3)	47 (14.7)		4 (18.2)	10 (14.9)	
4B	20 (20.6)	80 (25.1)		13 (59.1)	45 (67.2)	
4C	17 (17.5)	81 (25.4)		4 (18.2)	10 (14.9)	
5	49 (50.5)	111 (34.8)		1 (4.5)	2 (3.0)	
US-reported LN metastasis (%)	45 (46.4)	126 (39.5)	0.240	10 (45.5)	19 (28.4)	0.190

*HR* hormone receptor, *ER* estrogen receptor, *PR* progesterone receptor, *HER-2* human epidermal growth factor receptor 2, *LN* lymph node, *US* ultrasound, *BI-RADS* Breast Imaging Reporting and Data System, *IDC* invasive ductal carcinoma, *ILC* invasive lobular carcinoma.

### Radiomics feature extraction and selection

**Figure 3:**
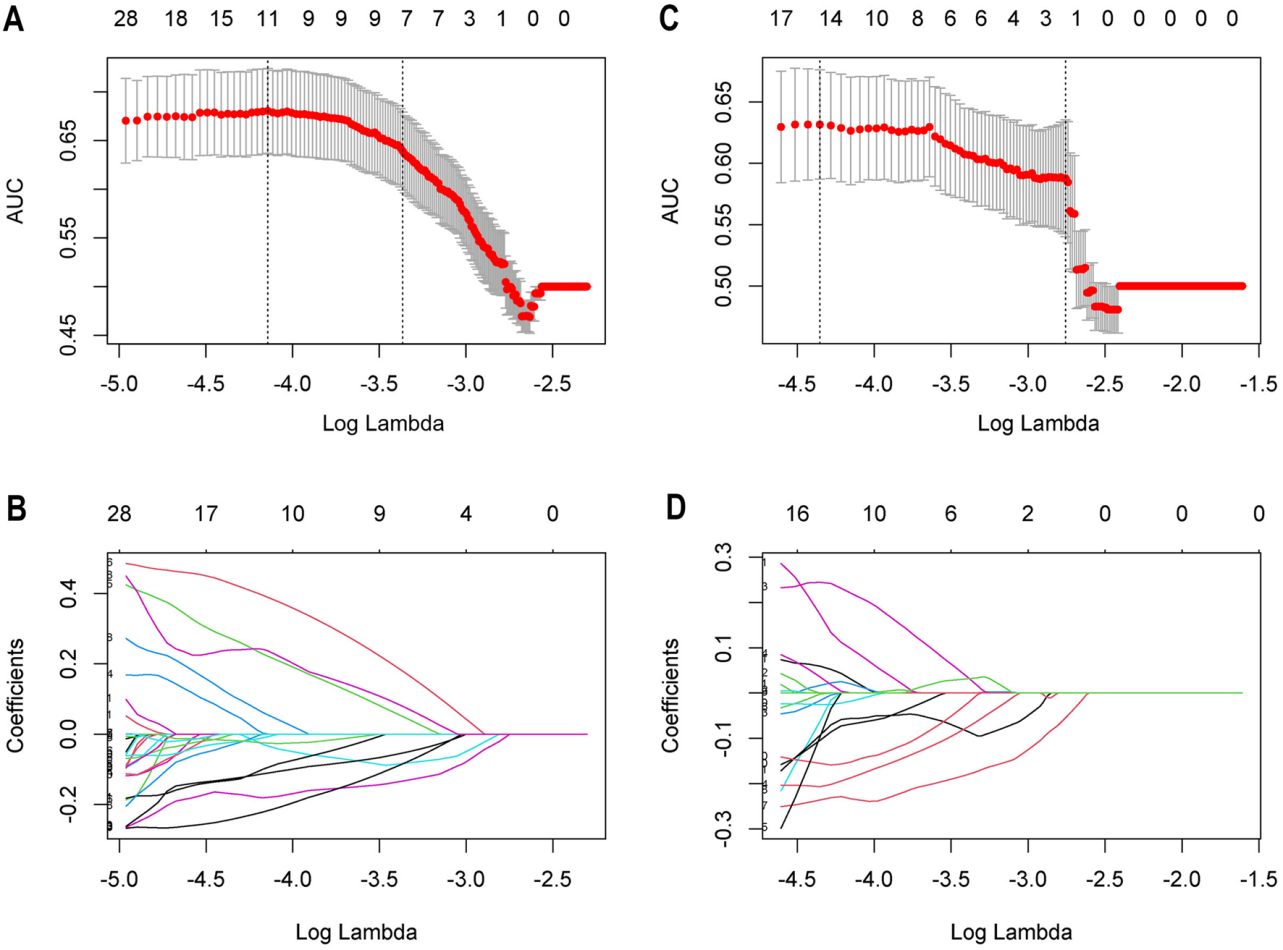
Radiomic feature selection using the least absolute shrinkage and selection operator (LASSO) logistic regression. The selection of tuning parameter (Lambda) in the LASSO model. (**A,C**) The area under the curve (AUC) was plotted versus log (Lambda). The dashed lines indicate the selected optimal log(Lambda) value and the location of one standard error. The optimal Lambda values of 0.015879 and 0.012864 with log (Lambda) of −4.142732 and −4.353298 were chosen according to tenfold cross-validation. (**B,D**) Graphs of variation of the radiomics characteristic coefficients with log(Lambda) for long-axis and short-axis ultrasound planes, respectively. *AUC* area under the curve.

Our radiomics analysis involved the extraction of a number of features, comprising 765 features from long-axis images and 754 features from short-axis images, which were carefully selected based on their ICCs > 0.75. This selection process ensured the inclusion of only the most stable and reliable radiomics features in our analysis. To further refine our feature selection, the Mann–Whitney U test was applied. This step led to the identification of 155 long-axis features and 165 short-axis features with p-values < 0.05. These features were subsequently selected for further investigation. Using the LASSO method (Fig. 3), we narrowed down our selection to 11 significant features derived from long-axis images and 15 features extracted from short-axis images. Detailed information on these selected features can be found in Table 3.

**Table 3:** Radiomics feature selection results.

Ultrasound section	Radiomics features	Coefficient
Long-axis	original_glszm_LargeAreaLowGrayLevelEmphasis	0.05471162
	original_glszm_LowGrayLevelZoneEmphasis	−0.033897205
	original_glszm_SmallAreaLowGrayLevelEmphasis	−0.094685486
	wavelet.LLH_glrlm_RunLengthNonUniformityNormalized	−0.179917998
	wavelet.LHH_glszm_SmallAreaLowGrayLevelEmphasis	−0.217623976
	wavelet.HLH_firstorder_Mean	0.221558923
	wavelet.HLH_glszm_SmallAreaLowGrayLevelEmphasis	−0.006933122
	wavelet.HHL_glszm_SmallAreaHighGrayLevelEmphasis	−0.110693721
	wavelet.HHH_glrlm_HighGrayLevelRunEmphasis	0.389062391
	wavelet.HHH_glszm_SmallAreaHighGrayLevelEmphasis	−0.025594806
	wavelet.LLL_glszm_HighGrayLevelZoneEmphasis	0.236738394
Short-axis	original_glcm_Imc2	0.05938874
	original_glszm_GrayLevelNonUniformityNormalized	−0.23621553
	original_gldm_DependenceNonUniformityNormalized	−0.063225483
	original_gldm_LargeDependenceHighGrayLevelEmphasis	0.244358475
	wavelet.LHH_glszm_LargeAreaHighGrayLevelEmphasis	−0.093705346
	wavelet.LHH_glszm_SizeZoneNonUniformityNormalized	−0.206875877
	wavelet.LHH_glszm_SmallAreaHighGrayLevelEmphasis	−0.002356957
	wavelet.HLL_glcm_JointAverage	0.016693034
	wavelet.HLL_glcm_Imc2	0.035923641
	wavelet.HLL_gldm_LowGrayLevelEmphasis	−0.104822439
	wavelet.HHL_glszm_LargeAreaEmphasis	−0.15513079
	wavelet.LLL_glcm_MaximumProbability	−0.016818457
	wavelet.LLL_glszm_LargeAreaHighGrayLevelEmphasis	−0.025320527
	wavelet.LLL_glszm_LargeAreaLowGrayLevelEmphasis	0.174228909
	wavelet.LLL_glszm_ZonePercentage	−0.097968346

### Long-axis and short-axis models

Based on the selected features, we developed separate long-axis and short-axis models. These models were instrumental in computing Rad-scores for each patient within the discovery and validation cohorts. In the discovery cohort, it is noteworthy that the long-axis model exhibited a marginally lower AUC value compared to the short-axis model. However, statistical analysis using the Delong test revealed no significant difference between the two models (p = 0.821). Contrarily, in the validation cohort, the long-axis model demonstrated a higher AUC value compared to the short-axis model (Delong test, p = 0.528); however, this difference was not statistically significant.

**Figure 4:**
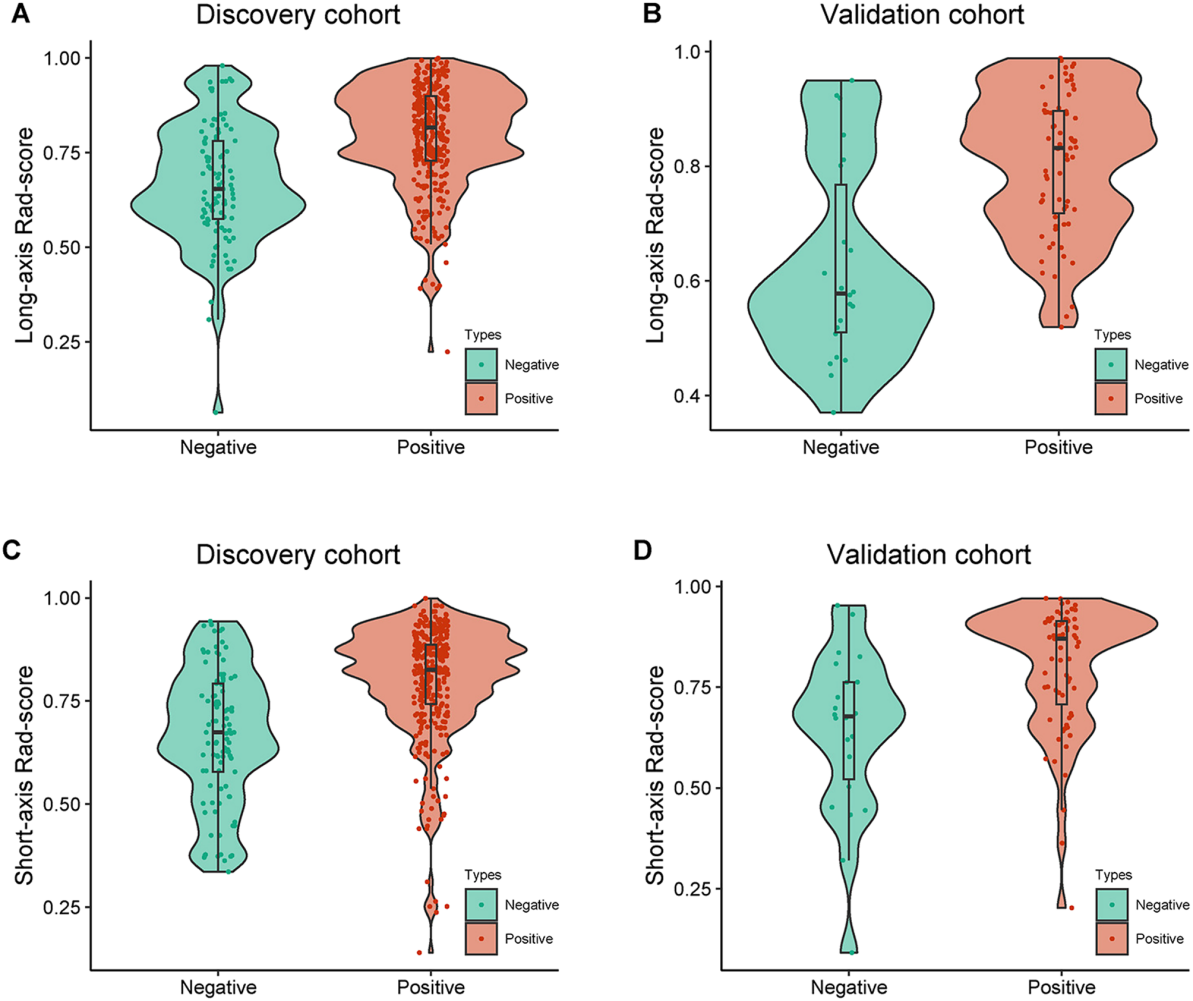
The distribution of radiomics scores (Rad-scores). Long-axis Rad-scores for each patient across both hormone receptor (HR) positive and HR negative breast cancers, as well as the distribution of short-axis Rad-scores, are assessed in both the discovery cohort (**A,C**) and the validation cohort (**B,D**), respectively. *Rad-score* radiomics score.

For a comprehensive visual comprehension, the distributions of the long-axis and short-axis Rad-scores, along with HR status, are presented in Fig. 4. Importantly, significant differences were observed in the distribution of Rad-scores for HR positive and HR negative patients in both the discovery and validation cohorts, with all p-values < 0.05, as highlighted in Table 4.

**Table 4:** Rad-scores for the discovery and validation cohorts.

Model	Cohort	Rad-score		p
		HR positive	HR negative	
Long-axis	Discovery	0.816 (0.729, 0.899)	0.654 (0.575, 0.781)	< 0.001
	Validation	0.831 (0.718, 0.896)	0.578 (0.510, 0.768)	< 0.001
Short-axis	Discovery	0.826 (0.742, 0.887)	0.674 (0.578, 0.792)	< 0.001
	Validation	0.871 (0.708, 0.915)	0.678 (0.522, 0.763)	< 0.001

*Rad-score* radiomics score, *HR* hormone receptor.

### Clinical risk factors

Our univariate analysis found that US-reported tumor size held predictive potential as a clinical risk factor. The obtained result implies that tumor size may possess a more robust predictive capacity for HR status compared to other clinical factors. Based on the tumor size variable, we developed a regression prediction model. The results indicated that, for the discovery cohort, the model achieved an AUC of 0.592, with a sensitivity of 0.571 and a specificity of 0.598. For the validation cohort, the model achieved an AUC of 0.657, with a sensitivity of 0.701 and a specificity of 0.591.

### Combined model development and assessment

To harness the full predictive power of our radiomics analysis and clinical risk factor, we constructed a combined model. This model integrated the long-axis Rad-score, short-axis Rad-score, and tumor size. The combined model was developed using the discovery cohort and subsequently validated using the validation cohort. The results were promising, with the combined model yielding AUC values of 0.788 and 0.822 in the discovery and validation cohorts, respectively. These outcomes were significantly better than those obtained from the model based solely on tumor size. These values surpassed those of the individual long-axis and short-axis models, suggesting the potential synergy of radiomics and clinical information.

Statistical analysis using Delong test revealed that the AUC value of the combined model demonstrated a statistically significant difference compared to the long-axis model (p = 0.010). However, there was no statistically significant difference when compared to the short-axis model in the discovery cohort (p = 0.073).

In the validation cohort, the combined model demonstrated no statistically significant differences compared to the long-axis and short-axis models (p = 0.515 and p = 0.399, respectively). Moreover, a comprehensive assessment of the precision, sensitivity, and specificity of the three models was performed, as detailed in Table 5.

**Table 5:** Prediction performance of long-axis, short-axis, and combined models in the discovery and validation cohorts.

Model	Cohort	AUC (95% CI)	Specificity	Sensitivity	Accuracy
Long-axis	Discovery	0.743 (0.686–0.801)	0.629	0.790	0.752
	Validation	0.795 (0.665–0.925)	0.636	0.940	0.865
Short-axis	Discovery	0.751 (0.695–0.808)	0.722	0.702	0.707
	Validation	0.744 (0.623–0.865)	0.909	0.552	0.640
Combined	Discovery	0.788 (0.738–0.838)	0.794	0.671	0.700
	Validation	0.822 (0.713–0.932)	0.773	0.761	0.764

*AUC* area under the curve, *CI* confidential interval.

**Figure 5:**
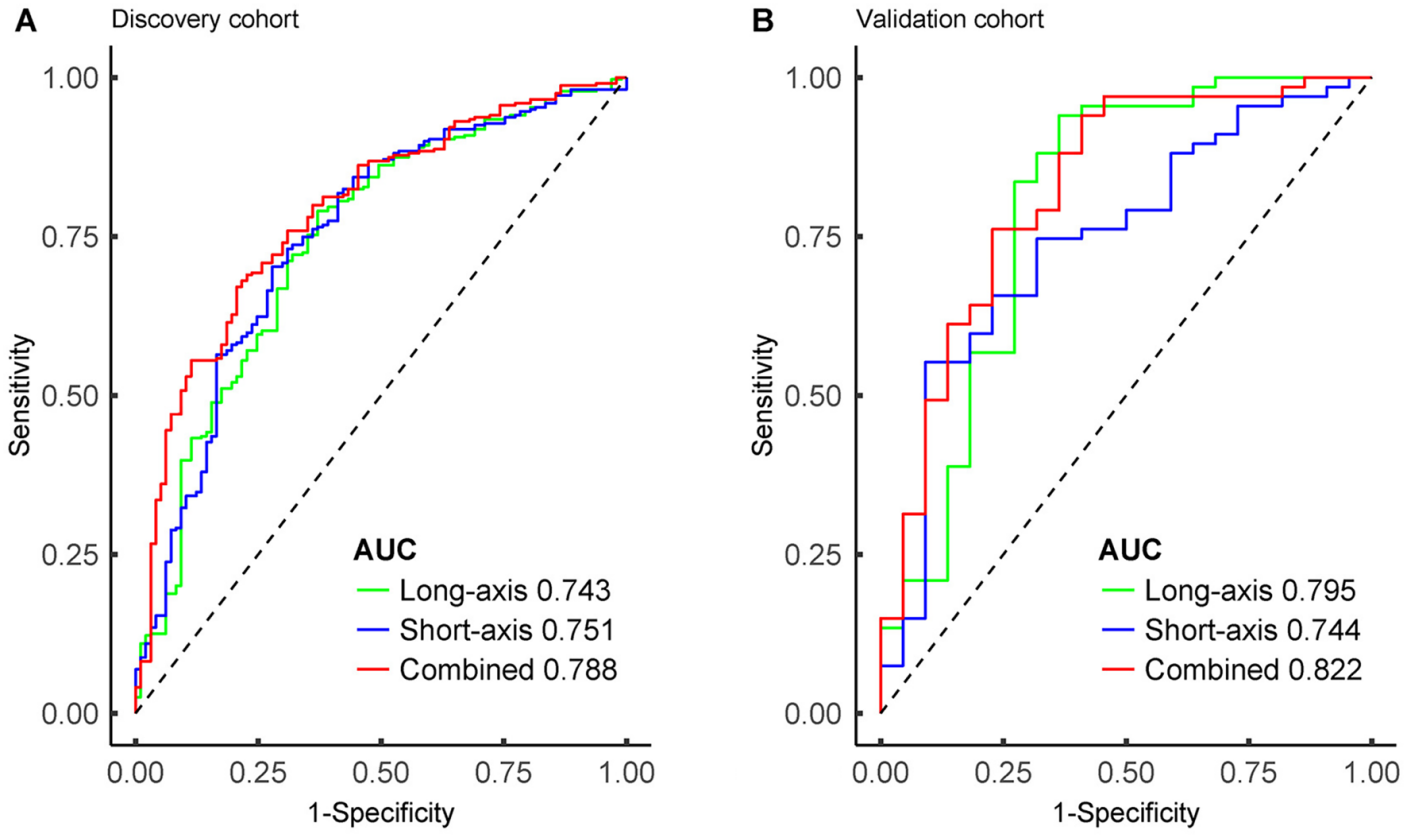
Performances for hormone receptor (HR) positive prediction. Receiver operating characteristic curves were generated for the long-axis model, short-axis model, and combined model in both the discovery (**A**) and validation (**B**) cohorts. *AUC* area under the curve.

To illustrate the discriminative efficacy of our models, ROC curves were generated, delineating the capacity to distinguish HR status in both the discovery and validation cohorts. These curves are depicted in Fig. 5, emphasizing the improved performance of the combined model.

### Clinical application

**Figure 6:**
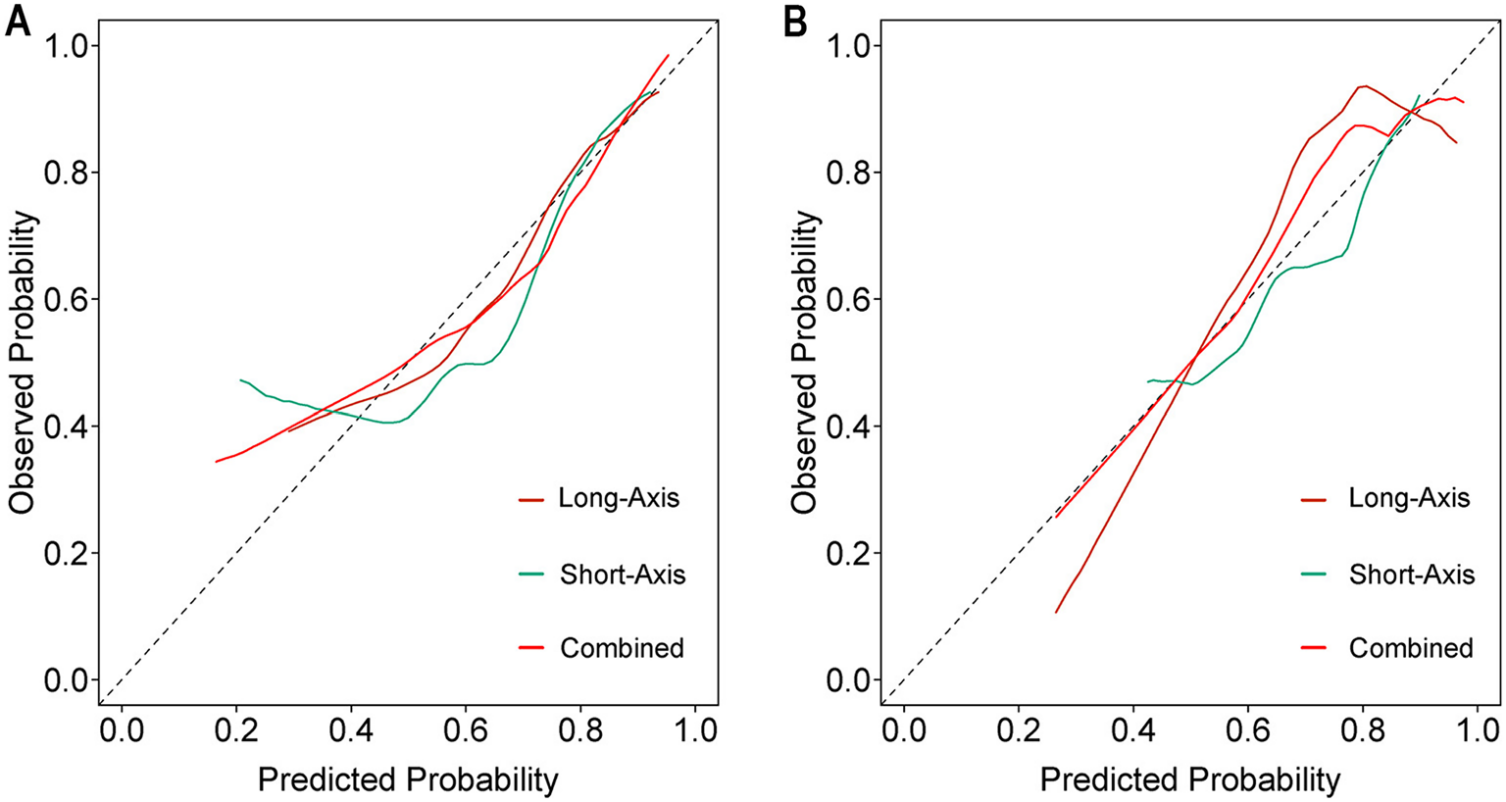
Calibration curves of the three models. Calibration curves constructed for the long-axis model, short-axis model, and combined model within both the discovery (**A**) and validation (**B**) cohorts. These curves described a good fitness between predicted and observed outcomes for hormone receptor (HR) positive and HR negative breast cancers across all three models. The ideal prediction was depicted by the gray line. A closer fitness to the gray line represented a well-calibrated model.

**Figure 7:**
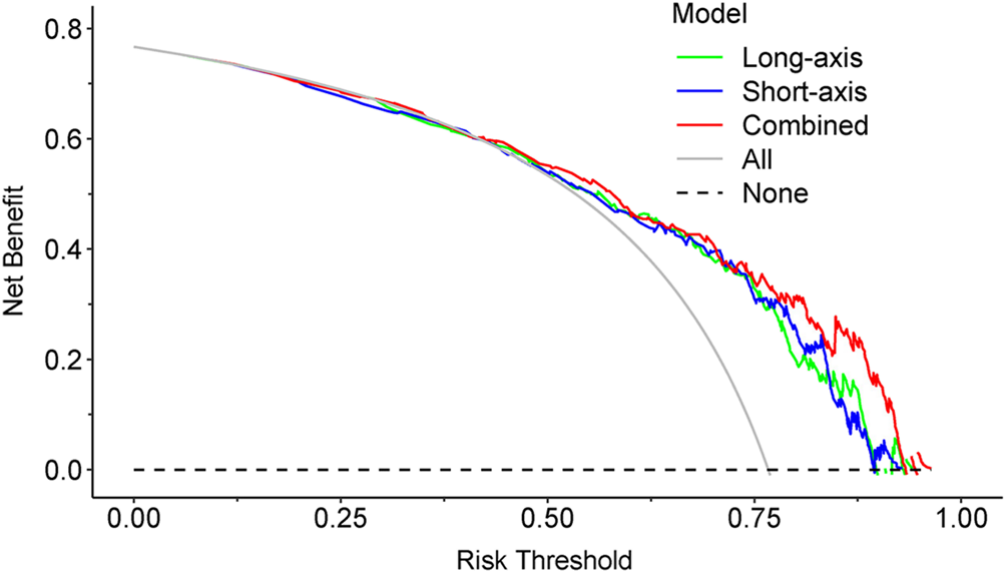
Decision curve analyses for the three models. Decision curves were generated for the long-axis model, short-axis model, and combined model. The combined model exhibited a superior net benefit compared to the other two models across the majority of threshold probabilities.

In the practical application of our findings, we conducted calibration curves, as depicted in Fig. 6, for the long-axis, short-axis, and combined models. These curves ensure that our models are well-calibrated and suitable for clinical use. The Hosmer–Lemeshow test further confirmed the satisfactory fit of our models. Moreover, decision curve analyses were conducted to assess the clinical practicability of the three models, as shown in Fig. 7. These analyses demonstrate the net benefit of employing our models in clinical decision-making, further underscoring their utility.

**Figure 8:**
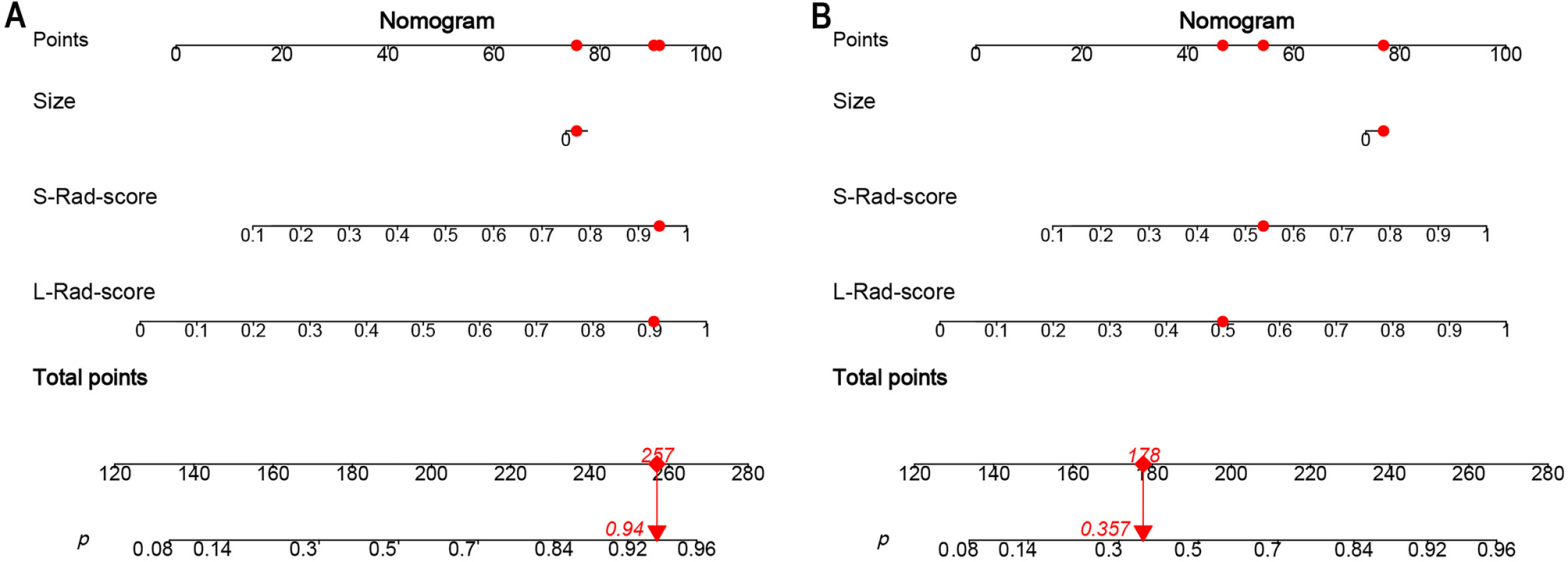
Combined model nomograms. Nomograms were constructed by integrating the long-axis radiomics score (Rad-score), short-axis Rad-score, and tumor size to differentiate between hormone receptor (HR) positive and HR negative breast cancers. Patient 1 (**A**) was a 72-year-old female diagnosed with a 3.0-cm-sized breast lesion. Her long-axis and short-axis Rad-scores were 0.907 and 0.943, respectively. The total score calculated was 257 points, corresponding to a high probability of HR positivity (0.940). Pathological examination confirmed HR positive breast cancer in this patient. Patient 2 (**B**) was a 47-year-old female diagnosed with a 5.0-cm-sized breast lesion. Her long-axis and short-axis Rad-scores were 0.499 and 0.537, respectively. The total score obtained was 178 points, indicating a lower probability of HR positivity (0.357). Pathological analysis confirmed HR negative breast cancer in this case. *L-Rad-score* long-axis radiomics score, *S-Rad-score* short-axis radiomics score.

In Fig. 8, we illustrate two examples showing the clinical application of our model. These nomograms serve as practical tools for estimating hormone receptor (HR) status in breast cancer patients. They incorporate factors such as the long-axis Rad-score, short-axis Rad-score, and tumor size, providing valuable insights for clinical decision-making.

## Discussion

This study aimed to explore the potential of US radiomics and clinical characteristics in predicting HR status in breast cancer patients. We selected radiomics features for stability and significance, and developed long-axis and short-axis models. We found that ultrasound-reported tumor size emerged as a clinical risk factor for HR status prediction. Furthermore, we integrated long-axis Rad-score, short-axis Rad-score, and tumor size into a combined model that outperformed individual models, achieving an AUC of 0.788 in the discovery cohort and 0.822 in the external validation cohort. Our models demonstrated well-calibrated performance suitable for clinical use, with decision curve analyses confirming their utility. We also presented a practical nomogram to aid clinicians in estimating HR status. These findings suggest that long-axis and short-axis radiomics features, when combined with tumor size, can enhance HR status prediction, potentially improving personalized treatment strategies for breast cancer patients.

Several studies have investigated the role of radiomics in breast cancer. Yu et al.^23^ established a radiomics model using machine learning to predict axillary lymph node metastasis through the analysis of ultrasound images, achieving a moderate AUC of 0.71. Romeo and colleagues^24^ constructed a prediction model based on radiomics features and machine learning applied to ultrasound imaging. This model exhibited the ability to discriminate between benign and malignant breast cancers, yielding a noteworthy AUC value of 0.82. However, in these investigations, there has been a notable emphasis on a singular imaging plane, particularly along the long axis. This raises the inquiry as to whether relying solely on a singular ultrasound plane inherently results in a reduced acquisition of radiomics information. In addressing this concern, our study systematically examined both long-axis and short-axis models, aiming to comprehensively evaluate potential disparities in imaging radiomics information linked to each orientation. We found that both orientations yielded comparable results, offering clinicians flexibility in their choice of imaging orientation for radiomics-based HR status prediction. However, the performance of single ultrasonic plane model is insufficient (AUC < 0.80), suggesting that combining the long-axis and short-axis features with clinical information might be necessary for achieving a higher predictive value.

Then, we developed a combined predictive model that demonstrated superior performance in predicting HR-positive breast cancer. The superior performance of our combined model is likely due to the integration of the long-axis and short-axis radiomics features, which show significant differences between HR-positive and HR-negative breast cancers. This suggests that capturing a comprehensive range of radiomics features from different planes can effectively predict the HR expression of breast cancer. This enhancement also may be attributed to the comprehensive insights into breast tumor morphology and structure provided by long-axis and short-axis perspectives, allowing for a more thorough understanding of the spatial distribution of biological features^19–21^. The comprehensive nature of information from both planes, along with the model's ability to capture diverse characteristics and variations, may contribute to increased robustness and adaptability, ultimately enhancing the overall predictive performance.

Previous literature reported that clinical features of breast cancer, such as tumor size and age, were associated with HR status^25,26^. In a study by Krizmanich-Conniff et al., it was demonstrated that triple receptor-negative cancer often appears as a hypoechoic or complex mass with an irregular shape and noncircumscribed margins on ultrasound and is more common in younger women with higher pathological grades^27^. In this study, we found significant difference between HR positive and HR negative groups in the tumor size of the breast cancer lesions using univariate logistic regression. This may be related to HR negative tumors, characterized by higher cell proliferation rates and possibly unique tissue structures, may contribute to larger apparent diameters^26^. Additionally, the propensity for HR negative tumors to exhibit lymph node involvement could further influence local tumor spread, impacting the overall tumor size.

We derived four distinct categories of radiomics features from ultrasound images, comprising shape-based, first-order statistical, texture, and wavelet features. The predominant elements integrated into the model were primarily texture and wavelet features. The utilization of wavelet features facilitates the computation of image signal resolution across various temporal, spatial, and frequency scale planes^28,29^. Texture analysis, a pivotal facet of this methodology, streamlines the extraction and quantification of intricate details, including regularity, roughness, and grey-level attributes of lesions, which might evade visual detection^30,31^. This approach affords a more comprehensive and nuanced portrayal of lesion characteristics. Consequently, our research demonstrates the critical role of texture and wavelet features in predicting HR positive breast cancer. Although the radiomics features extracted from the long-axis and short-axis planes in ultrasound images differ, they predominantly consist of texture and wavelet features. Moreover, the models established for the long-axis and short-axis planes exhibit comparable predictive performance, with no statistically significant differences noted in the validation set. By further integrating the radiomics features from the long-axis and short-axis planes, the combined model enhances predictive performance, demonstrating the comprehensive nature of these two imaging perspectives.

Huang et al.^32^ utilized dynamic contrast-enhanced magnetic resonance imaging (DCE-MRI) to establish a radiomics signature for distinguishing luminal and non-luminal molecular subtypes in invasive breast cancer. Their radiomics signature, derived from the second phase of DCE-MRI images in 135 patients, demonstrated strong discrimination in both training (AUC = 0.86) and testing sets (AUC = 0.80), without identifying clinical risk factors as independent predictors. Sheng et al.^33^ employed machine learning techniques integrating MRI radiomics features and clinical data to predict the HR positive subtype in invasive ductal breast cancer. The eXtreme gradient boosting method exhibited superior performance with an AUC of 0.828 in the validation cohort, which included 190 women with various molecular subtypes. However, limitations of above studies included a small sample size, potentially introducing selection bias, and highlighting shortages of MRI, such as high cost and limited suitability for preoperative evaluation. In comparison, our multicenter study, involving 505 patients and external validation, confirmed the robustness of our model with an AUC of 0.822. Uniquely leveraging ultrasound imaging, our study offered advantages like the absence of contrast agents, easy equipment accessibility, and user-friendly operation, addressing some of the limitations associated with MRI-based approaches. The inclusion of both long-axis and short-axis ultrasound images provided a comprehensive tumor assessment, potentially enhancing our model's predictive performance. This comprehensive model contributes to the increased clinical applicability and credibility of our predictive model.

Gong et al.'s research^34^ has illustrated the potential of two-dimensional ultrasound radiomics in predicting HR positive in breast cancer. Their US radiomics model achieved an AUC value of 0.817, indicating the capability of US radiomics in predicting HR positive. However, Gong et al. utilized a single ultrasound plane radiomics approach with much smaller sample size of 120 lesions, achieving an AUC of 0.817. In contrast, our study included a significantly larger sample size and underwent rigorous external validation, resulting in a slightly improved AUC of 0.822. Despite the similarity in predictive performance between the two studies, our strength lies in the comprehensive approach adopted. We integrated both long-axis and short-axis planes, along with tumor size, thereby enhancing the accuracy and reliability of the predictive model. As a result, our research provides a more robust and clinically valuable tool for patients diagnosed with breast cancer.

Despite the promising results, our study has several limitations. Firstly, our study primarily relied on retrospective data, which may introduce selection bias. Prospective studies could provide more robust evidence. Secondly, the exclusion of non-mass tumors from our study may introduce some bias in patient selection. However, it's important to note that the absence of a standardized segmentation method for non-mass tumors contributed to this limitation. Thirdly, we did not incorporate other potential biomarkers, such as genomics or proteomics data, which could further refine HR status prediction models. Lastly, the segmentation of the tumor's ROI was not automated, resulting in a time-consuming and labor-intensive process. This challenge could potentially be addressed in the future by leveraging an artificially intelligent system.

## Conclusion

In conclusion, we have developed and validated a combined model for distinguishing between HR positive and HR negative breast cancers. This was achieved through the integration of key parameters, including the long-axis Rad-score, short-axis Rad-score, and tumor size. The superior performance exhibited by this combined model underscores its potential for tailoring individualized treatment for patients with invasive breast cancer. However, future research should prioritize the investigation of larger and more diverse cohorts and the validation of our model across multiple centers.

## Data Availability

The datasets used and/or analysed during the current study available from the corresponding author on reasonable request.
